# The impact of genes and environment assessed longitudinally on psychological and somatic distress in twins from ages 15 to 35 years

**DOI:** 10.1017/S0033291724003222

**Published:** 2025-02-06

**Authors:** Nathan A. Gillespie, Baptiste Couvy-Duchesne, Michael C Neale, Ian B. Hickie, Nicholas G Martin

**Affiliations:** 1 Virginia Institute for Psychiatric and Behaviour Genetics, Department of Psychiatry, Virginia Commonwealth University, Richmond, VA, USA; 2 QIMR Berghofer Medical Research Institute, Genetic Epidemiology Laboratory, Brisbane, Queensland, Australia; 3 Sorbonne University, Paris Brain Institute – ICM, CNRS, Inria, Inserm, AP-HP, Hôpital de la Pitié Salpêtrière, F-75013, Paris, France; 4 Institute for Molecular Bioscience, The University of Queensland, St Lucia, Queensland, Australia; 5 Brain and Mind Institute, University of Sydney, New South Wales, Australia

**Keywords:** anxiety, depression, gene, longitudinal, somatic, twin, psychological distress

## Abstract

**Background:**

Genetically informative twin studies have consistently found that individual differences in anxiety and depression symptoms are stable and primarily attributable to time-invariant genetic influences, with non-shared environmental influences accounting for transient effects.

**Methods:**

We explored the etiology of psychological and somatic distress in 2279 Australian twins assessed up to six times between ages 12–35. We evaluated autoregressive, latent growth, dual-change, common, and independent pathway models to identify which, if any, best describes the observed longitudinal covariance and accounts for genetic and environmental influences over time.

**Results:**

An autoregression model best explained both psychological and somatic distress. Familial aggregation was entirely explained by additive genetic influences, which were largely stable from ages 12 to 35. However, small but significant age-dependent genetic influences were observed at ages 20–27 and 32–35 for psychological distress and at ages 16–19 and 24–27 for somatic distress. In contrast, environmental influences were predominantly transient and age-specific.

**Conclusions:**

The longitudinal trajectory of psychological distress from ages 12 to 35 can thus be largely explained by forward transmission of a stable additive genetic influence, alongside smaller age-specific genetic innovations. This study addresses the limitation of previous research by exhaustively exploring alternative theoretical explanations for the observed patterns in distress symptoms over time, providing a more comprehensive understanding of the genetic and environmental factors influencing psychological and somatic distress across this age range.

## Introduction

Previous research by Gillespie, Kirk, et al. (Gillespie, Kirk, et al., 2004) on self-reported anxiety and depression symptoms from ages 20 to 70 years revealed a complex pattern of genetic influences across the lifespan. While genetic determinants were largely stable, there was evidence of age-specific genetic effects at different life stages. The study identified genetic effects at age 20, with additional genetic influences becoming apparent at later ages for some individuals. The current study seeks to extend these findings by examining a younger sample of twins while also testing competing theories to best explain the observed longitudinal stability of genetic influences on symptoms of anxiety and depression including somatic distress.

**Figure 1.**
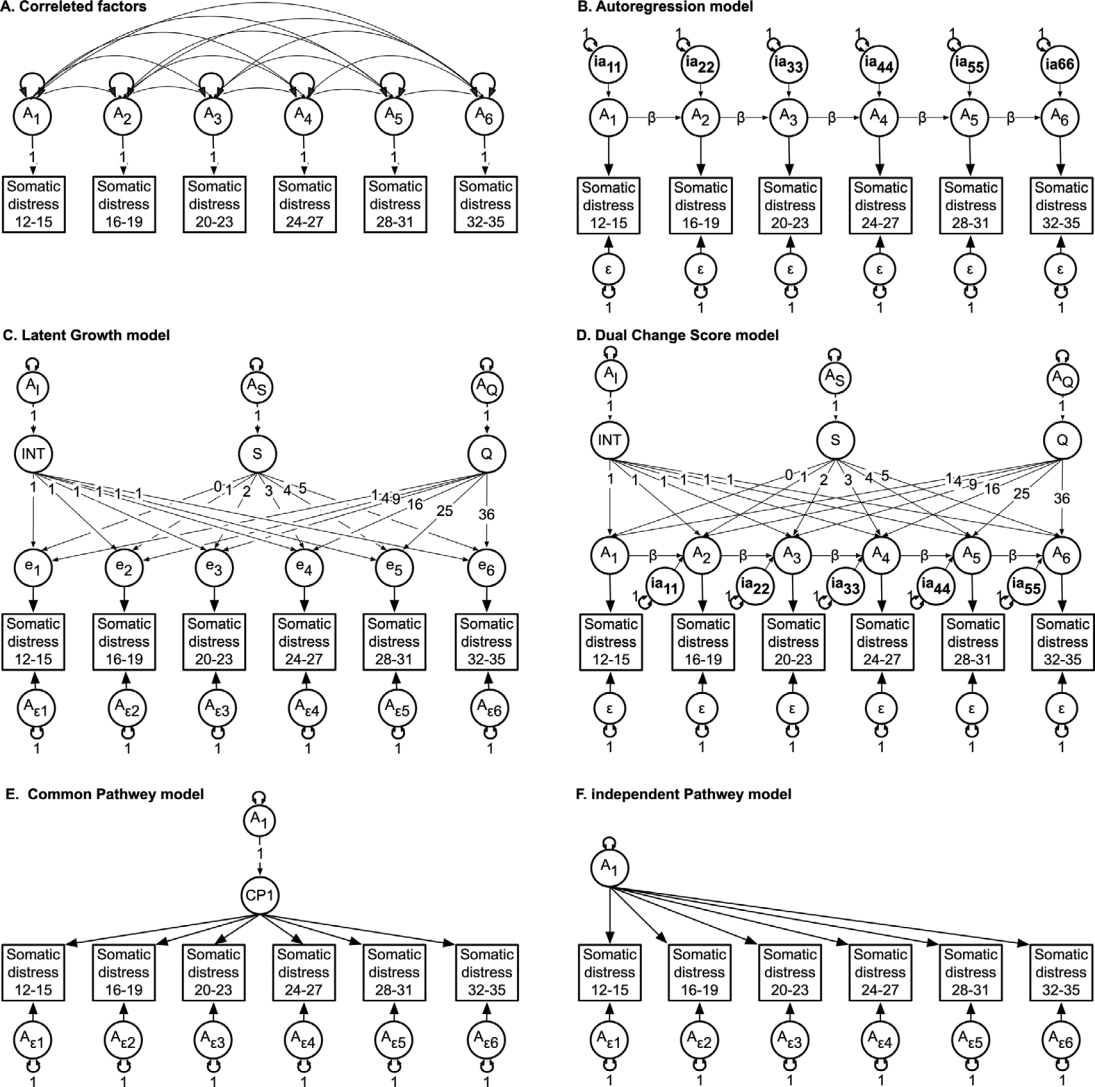
Multivariate correlated factors and the five competing hypotheses to explain the sources of variance-covariance between the SPHERE domain scores at each age interval. *Note:* For brevity, only additive genetic components and residuals are shown. **(A)** The correlated factors model (null hypothesis) is an atheoretical method for estimating the size of genetic (A1-A6) (and environmental) variance-covariances (double-headed arrows). **(B)** The autoregression model predicts causal process of inertial effects whereby genetic (or environmental) components at one time causally affect genetic variation at the next time e.g., A1 to A2 via β. This method also identifies age-dependent genetic innovations (ia11-ia66) and age-specific residual variances (ε). **(C)** The latent growth model predicts that stability and change in the variance-covariance structure and observed means are explained by latent intercept (INT), linear (S) and quadratic (Q) growth processes. The INT, S and Q factor variances are further decomposed into genetic (Ai, As and Aq) (and environmental) components. Genetic (Aε1-Aε6) and environmental residuals are also estimated. **(D)** The dual change score model attributes change to autoregression and latent growth processes. **(E)** The common pathway model predicts that variance-covariance is explained by one or more common pathways. **(F)** In the independent pathway model, genetic and environmental components are estimated independently.

Analyses of genetically informative twin data spanning childhood through to early old age have consistently found that individual differences in symptoms of anxiety and depression are stable and largely attributable to time invariant additive genetic influences, whereas non-shared environmental influences account for short-term variance (D.I. Boomsma et al., 2005; Nivard et al., 2015). Typically, these analyses have relied on autoregression models (see Figure 1), which predict that longitudinal correlations are determined by random, time-specific genetic and environmental effects that are more or less persistent over time (Boomsma et al., 1989; Boomsma & Molenaar, 1987; Eaves et al., 1986; Guttman, 1954). An advantage of autoregression models is their utility for highlighting inertial effects, whereby latent influences at one time are assumed to be causally related to immediately preceding latent influences including new latent inputs, also known as innovations. This method has been typically applied to investigate both age-invariant and age-dependent sources of individual differences. For example, genetically informative autoregression analyses based on early childhood data have revealed that genetic influences on the symptoms of anxiety and depression are dynamic and age-dependent (Kendler et al., 2008; Nivard et al., 2015). In addition to anxiety and depression (Gillespie et al., 2015; Gillespie, Kirk, et al., 2004), this type of modeling has been applied, but not limited to, studies of personality (Gillespie, Evans, et al., 2004), substance use (Long et al., 2017), brain aging (Gillespie, Hatton, et al., 2022), and recently to BMI data (Cardon, 1995; Cornes et al., 2007; Gillespie, Gentry, et al., 2022).

A limitation of the literature describing applications of autoregression modeling to anxiety and depression is the absence of alternative hypothesis testing. We have argued elsewhere (Gillespie et al., 2015) that different models for the roles of genes and environment in development will lead to different predictions regarding the correlational patterns in longitudinal data from family members.

For example, the variances and covariances of longitudinal measures of depression in twin data could depend on individual genetic or environmental differences in inherent growth patterns unfolding with age, that is, ‘random growth curves’ (Duncan et al., 1994; Duncan & Duncan, 1991; McArdle, 1986; McArdle & Epstein, 1987; Nesselroade & Baltes, 1974) (see Figure 1C). Thus, in twin-based latent growth curve models, genetic and environmental influences are predicted to determine initial levels and rates of change over time.

As illustrated in Figure 1D, different developmental processes could exist for the genetic and environmental components of developmental change. For example, it is conceivable that genetic influences may account for individual differences in levels and rates of change, whereas autoregressive effects could account for the ‘remembering’ or ‘forgetting’ of time-dependent non-genetic influences. Indeed, this innovative approach of combining the features of autoregression (Eaves et al., 1986) with latent growth models (McArdle, 1986; McArdle, 1994; Nesselroade & Baltes, 1974), known as the dual change score model, is mathematically and statistically equivalent to certain random coefficient, multilevel or hierarchical linear models (Bryk & Raudenbush, 1987; McArdle et al., 1991; McArdle & Hamagami, 1992; Mehta & West, 2000; Miyazaki & Raudenbush, 2000) and has been applied to cognition (McArdle, 2001; McArdle & Hamagami, 2001) drug availability (Gillespie et al., 2007), alcohol use (Long et al., 2017), and symptoms of depression (Gillespie et al., 2015; Hishinuma et al., 2012). In our longitudinal analysis of symptoms of depression in boys and girls aged 8–18 years, we found that genetic and environmental correlations were explained by distinct mechanisms, that is, growth and autoregression processes, respectively. However, apart from Gillespie et al. (Gillespie et al., 2015), we are unaware of other instances of genetically informative dual change score models applied to longitudinal assessment of anxiety and depression.

It is also conceivable that genetic and environmental influences observed across time are time-invariant and better explained by common or independent genetic and environmental pathway theories (Kendler et al., 1987; McArdle & Goldsmith, 1990). The common pathway model predicts that longitudinal covariance is explained by one or more common factors (directly analogous to a factor analysis) which in turn can be decomposed into genetic and environmental influences. In contrast, and as the name suggests, the independent pathway model predicts that longitudinal covariance is explained by separate genetic and environmental influences acting independently (see Figure 1F). To our knowledge, no previous genetically informative studies have tested the fit of these models to longitudinal depression or anxiety data.

### Aim

Our aim, therefore, was to explore the etiology of symptoms of psychological distress (comprising non-clinical anxiety and depression items) and somatic distress in a longitudinal assessment of men and women aged 12–35 years. In addition to estimating the stability of heritable individual differences, we tested competing hypotheses to explain best the longitudinal variation in genetic and environmental influences over time.

## Materials and methods

### Subjects

Discussed in detail elsewhere (Gillespie et al., 2013; Mitchell et al., 2019; Mitchell et al., 2020; Wright & Martin, 2004), subjects were monozygotic (MZ) and dizygotic (DZ) twins from the ongoing Brisbane Longitudinal Twin Study (BLTS) at the Queensland Institute of Medical Research (QIMR). The BLTS began in 1992 when twins were recruited from primary and secondary schools in the greater Brisbane area via media appeals and by word of mouth. To date, BLTS subjects have participated in six waves of assessment at average ages 12.2 (SD = 0.2, range = 12.0, 13.7), 14.2 (SD = 0.2, range = 13.6, 14.9), 16.3 (SD = 0.4, range = 16.0, 19.4), 18.1 (SD = 3.0, range = 12.3, 25.8), 25.0 (SD = 3.7, range = 18.5, 35.9), and 29.9 (SD = 4.2, range = 22.6, 38.7) years.

### Ethics

All BLTS assessment protocols were approved by the QIMR Berghofer Medical Research Institute-Human Research Ethics Committee. Written informed consent was obtained from all subjects.

### Phenotypic data

The Somatic and Psychological HEalth REport (SPHERE) is a 34-item assessment of common symptoms of mental distress and persistent fatigue by self-report (Hickie et al., 2001). Items were selected, tested, and validated from four widely used clinical assessments of mental health, based on their predictive ability (Hickie et al., 2001). Anxiety and depression items were selected from the General Health Questionnaire (Goldberg, 1978), chronic fatigue from the Schedule of Fatigue and Anergia (Hadzi-Pavlovic et al., 2000), neurasthenia from the Illness, Fatigue and Irritability Questionnaire (Hadzi-Pavlovic et al., 1997), and somatization items from the Diagnostic Interview Schedule (DSM)-III-R (American Psychiatric Association, 1987).

In this study, we analyzed the 12 items from the SPHERE-12, a shortened version of the original SPHERE, which were administered at all 6 BLTS assessments (see Supplementary Information S1). The SPHERE-12 consists of two domains: psychological distress and somatic distress. The psychological distress domain comprises six items assessing symptoms of anxiety and depression, including ’Feeling unhappy and depressed’ and ’Feeling nervous or tense’. The somatic distress domain includes six items measuring physical symptoms, such as ’Muscle pain after activity’ and ’Prolonged tiredness after activity’. Subjects were asked to respond to the prompt, ‘Over the past few weeks have you been troubled by…?’ for each item. Responses were recorded on a 3-point ordinal scale: 0 ‘never or some of the time’; 1 ‘a good part of the time’; and 2 ‘most of the time’. Domain scores were calculated by summing the respective item scores.

The SPHERE-12 has demonstrated predictive validity for DSM diagnoses, showing significant correlations with disability measures and doctors’ risk ratings (Hickie et al., 2001). Internal consistency was high, with Cronbach’s alpha of 0.90 for psychological items and 0.80 for somatic items (Hickie et al., 2001). Finally, test–retest reliability over 3–6 months was also high, with intraclass correlations of 0.81 and 0.80 for psychological and somatic scores, respectively (Hickie et al., 2001).

Given the variation in chronological age at each wave and unequal time intervals between the six waves, longitudinal analysis of these wave-based data will preclude meaningful understanding of age-related changes. For instance, ignoring irregular spacing between time intervals in longitudinal modeling can lead to biased parameter estimates (Estrada & Ferrer, 2019). Our solution, therefore, was to convert the wave-based data to age-based or age interval data. To reduce sparse data while maintaining computational efficiency, our age-anchored SPHERE scores were re-coded to one of the seven age intervals according to each individual’s age at the time of each BLTS assessment: 12–15; 16–19; 20–23; 24–27; 28–31; 32–35, and 36–39 years. Thus, for example, if two subjects ‘Subject A’ and ‘Subject B’ were each 20 years old at the 4^th^ and 5^th^ BLTS assessments, respectively, their SPHERE scores would be assigned to the 20 to 23 age interval. Based on comparisons using the software package bestNormalize in R_3.4.1_ (R Development Core Team, 2018), which attempts to find and execute the best normalizing transformation, domain scores at each wave were square root transformed (see Supplementary Figures S1 and S2).

Supplementary Table S1 includes summary statistics for the psychological and somatic distress domain score. Given the small sample size at ages 36–39, we restricted all subsequent analyses to the first five age intervals, that is, ranging 12–35 years. No subject provided data at all five age intervals. There were *N* = 1639, *N* = 972, *N* = 931, and *N* = 229 subjects with SPHERE scores at one, two, three, and four age intervals, respectively.

### Statistical analyses

We use the OpenMx2.21.11 software package with its raw data Full Information Maximum Likelihood (FIML) fit function (Boker et al., 2011) in R3.4.1 (R Development Core Team, 2018) t to test the assumptions (see below) of mean and variance homogeneity, that is, that there are no substantial differences between twin 1 and twin 2 with zygosity, nor differences between the means and variances of MZ and DZ twins (Neale & Cardon, 1992).

The raw data FIML option has several advantages. FIML is generally robust to moderate violations of normality under certain conditions (e.g., larger sample sizes, mild to moderate non-normality), particularly when compared to methods that apply listwise deletion (Enders, 2001). Moreover, FIML enables the inclusion and analysis of missing or incomplete data (see Supplementary Table S1 for the numbers of complete and incomplete twin pairs). Enders and Bandalos (Enders & Bandalos, 2001) have empirically demonstrated that FIML leads to increased parameter estimate efficiency compared to listwise deletion. These properties of FIML – its relative robustness to non-normality and its ability to handle missing data – contribute to more precise parameter estimates in many practical scenarios.

In all univariate and multivariate analyses, the mean of each variable was adjusted for the effects of sex using the definition variable option in OpenMx (Boker et al., 2011). By combining data from men and women and adjusting for sex differences, our aim was to interpret longitudinal covariance that is shared or common across the sexes. This approach allows us to identify patterns that are generalizable across sex, thus providing a foundation for understanding broader trends in the population.

### Mean and variance homogeneity

Each observed, phenotypic variable is assessed as four distinct measurements: MZ twin 1, MZ twin 2, DZ twin 1, and DZ twin 2. Models for twin data usually predict that the means and variances are the same across all (four) instances. Therefore, we began by testing these predictions of (i) equal means and (ii) equal variances across twin 1 and twin 2 within each zygosity group. These tests were followed by tests for mean and variance equality across zygosity.

### Twin pair correlations

Prior to the univariate model fitting, we estimated twin pair correlations by zygosity for the SPHERE domain scores at each age interval (Supplementary Table S1). If familial aggregation in a complex trait exists and is entirely attributable to shared family environments, then two expectations should hold: (1) both MZ and DZ twin pair correlations are statistically significant and greater than zero; and (2) the MZ and DZ twin pair correlations should not be significantly different from each other. If familial aggregation is attributable to shared environmental factors, both MZ and DZ correlations should be significantly greater than zero and not significantly different from each other at standard significance levels (e.g., *α* = 0.05). We note that if both correlations are not significantly different from zero, this would suggest a lack of familial aggregation altogether, rather than aggregation stemming from shared environmental factors.

### Univariate analyses

Univariate models were fitted to confirm familial aggregation by estimating the size, significance and relative contribution of genetic and environmental variance influences in each SPHERE domain score at each age. Specifically, we applied the Classical Twin Design (CTD), which relies on twins reared together, to decompose the total variation in each SPHERE domain score into additive (A) genetic variance, shared or common environmental (C), and non-shared or unique (E) environmental variance components (see Supplementary Figure X). This decomposition is achieved by exploiting the expected genetic correlations between MZ and DZ twin pairs; MZ twin pairs are genetically identical, whereas DZ twin pairs share, on average, only half of their genes. Therefore, MZ and DZ twin pair correlations (*r*_A_) for additive genetic effects are fixed to 1.0 and 0.5, respectively. The CTD assumes neither genotype by environmental interactions nor genotype-environment correlations and that parental mating is random. It also assumes that shared environmental effects are equal for MZ and DZ twin pairs, that is, equality of parental treatment, equality of environmental exposure, and no effects caused by placentation (Scarr, 1968). Given this equal environmental assumption, the MZ and DZ twin correlations (rC) for shared or common (C) environmental influences are both fixed to 1.0. Because all non-shared environmental influences (E), which include measurement error, are by definition uncorrelated, the MZ and DZ twin pair correlation (rE) for these ‘E’ influences is fixed to zero.

In the CTD, shared environmental (C) and non-additive genetic (D) influences are negatively confounded and cannot be modeled simultaneously (Martin et al., 1978b). The C variance estimate effectively combines shared environmental (C) and non-additive genetic (D) sources of variation. While DZ correlations less than half the MZ correlations in some age ranges suggested genetic non-additivity, we retained the C parameter because this pattern was not consistent across all ages. In our variance components analysis, the C estimate captures both environmental and non-additive genetic effects, with its sign indicating which influence predominates (positive for shared environment, negative for genetic non-additivity). Finally, because the SPHERE was assessed in a population-based sample of twins representing the full range of variation in psychological distress, the estimates of A, C, and E capture influences from both risk and protective factors across the population distribution.

### Multivariate analyses

The univariate method was extended to the multivariate to estimate the size and significance of genetic and environmental influences within and between the SPHERE domain scores across time. In order to have a reference for contrasting and choosing the best fitting theoretical model, we first fitted a multivariate ‘ACE’ correlated factors (see Figure 1A). As with a Cholesky Decomposition, the correlated factors reproduces the mean and variance-covariance information within and between variables while making no theoretical prediction regarding how genes and environments change over time. We chose the correlated factors model over the Cholesky decomposition because it typically yields less biased estimates of genetic and environmental influences, especially for multiple phenotypes (Verhulst et al., 2019). It also reduces false positive findings, is more robust to model misspecification, and offers more intuitive interpretations of the genetic and environmental correlations (Verhulst et al., 2019).

We then fitted competing models to explain the observed variance-covariance matrices between the longitudinal measures of somatic and psychological distress separately: autoregression, latent growth, dual change score, one common pathway (CP), two CPs, three CPs, and independent pathway models (see Figures 1B–1F). These seven models were selected as they represent a comprehensive range of theoretical perspectives on how traits may develop and change over time. The autoregression and latent growth models capture stability and change, respectively. The dual change score model combines elements of both. The common pathway and independent pathway models allow us to test different hypotheses about the underlying structure of genetic and environmental influences. By comparing these models, we aim to identify which theoretical framework best explains the longitudinal patterns of psychological and somatic distress in our sample.

The autoregression model predicts that time-specific random genetic or environmental effects may persist over time (autoregressive effects) (Eaves et al., 1986) (Figure 1B). As detailed in the Supplement, each observed phenotype can be decomposed into underlying genetic and environmental components, representing stable trait-like influences distinct from measurement error. At each occasion, these genetic and environmental components are determined by (i) new random genetic or environmental effects or ’innovations’ arising at that time point and (ii) the causal contribution, via the beta coefficients, from the components expressed at the preceding time. Such autoregression effects may occur between phenotypes or between the latent genetic and environmental components. Cross-temporal correlations within subjects emerge when autoregressive coefficients are non-zero, indicating that genetic or environmental innovations at one time point influence subsequent time points. These contributions may, under some circumstances, accumulate, potentially giving rise to developmental increases in genetic or environmental variance and increased correlations between adjacent measures. Depending on the magnitude of an innovation and its relative persistence, the observed variances and cross-temporal covariances may also increase during development towards a stable asymptotic value. Another feature of the autoregressive model is that cross-temporal correlations will tend to decay as a function of increasing time intervals. Note that our autoregression modeling included occasion-specific residual variance (E1 _res_–E4_res_) or random error components that include measurement error not captured by the genetic and environmental autoregression processes. See Eaves et al. (Eaves et al., 1986) for graphical examples of an application to longitudinal cognitive data. See Supplement describing constraints to ensure model identification.

In contrast, the latent growth model posits that developmental change follows a trajectory characterized by initial levels (intercept) and rates of change over time, typically modeled as linear and quadratic functions (Figure 1C). This approach allows for the examination of both genetic and environmental influences on these growth parameters. These models can be conceptualized as a specialized form of factor analysis where the underlying constructs represent initial levels and patterns of change over time. Factor loadings in these models typically correspond to the timing of measurements, but can also be simplified for ease of interpretation. In our latent growth model, we used simplified factor loadings of 0, 1, 2, 3, 4, and 5 for the linear change factor, corresponding to our six measurement occasions spanning ages 12–15, 16–19, 20–23, 24–27, 28–31, and 32–35 years, respectively. While this approach assumes equal intervals between measurements, it provides a reasonable approximation given the roughly equal spans of our age intervals. The intercept in this model represents the expected value at the first measurement (ages 12–15), and the slope represents the average change per measurement occasion. We acknowledge that this simplification may not capture the exact timing of developmental changes but allows for a straightforward interpretation of growth trajectories across the entire age range from adolescence to early adulthood. This approach allows us to model how individuals’ traits evolve, considering both their starting point and their rate of change. Mathematically, these loadings correspond to coefficients of *a priori* contrasts based on the measurement ages, enabling a nuanced analysis of developmental trajectories.

The dual change score model (Figure 1D) is a hybrid approach that combines elements of both the autoregressive and latent growth models. This model integrates the concepts of time-specific changes (as in the autoregressive model) with overall developmental trajectories (as in the latent growth model). By incorporating features from both approaches, the dual change score model allows for a more flexible representation of change over time, capturing both short-term fluctuations and long-term developmental trends.

The one common pathway (CP) model predicts that the covariance structure between all age intervals is explained by a single common liability, which can be decomposed into A, C, and E components (Figure 1E). Here, the size of the factor loadings from the CP indicate the strength of the relationship between the CP and the SPHERE domain scores at each age interval. We also explored the fit of the two and three CP models, to test the hypotheses that longitudinal covariance is accounted for by two and three correlated common pathways, respectively. In each case, residual variances not explained by the common pathway(s), or risks unique to each age interval, are decomposed into variable-specific genetic (A_ε1–6_) and environmental (C _ε1–6_ & E _ε1–6_) residuals. See Supplement describing constraints to ensure model identification.

Finally, the independent pathway model predicts that latent genetic and environmental risk factors separately or independently generate the observed covariance between the domain scores at each age interval (Figure 1F). Variances that are unique to each age interval (i.e., not captured by the independent pathway) again are decomposed into variable-specific genetic and environmental residuals.

### Model fit

For each univariate analysis, we determined the most likely sources of variance by fitting three nested sub-models in which the (i) C, (ii) A, and (iii) C and A influences were fixed to zero. Specifically, the significance of the A, C, and E parameters was determined using the change in the minus two Log-Likelihood (Δ-2LL), which under certain regularity conditions is asymptotically distributed as chi-squared with degrees of freedom equal to the difference in the number of free parameters in the two models. In multivariate analyses, we compared the fit of the competing models (Dual Change Score, Latent Growth, Autoregression, Common and Independent Pathway models) to the saturated variance components model, by again, relying on the change in Δ-2LL. For all univariate and multivariate analyses, the determination of the best-fitting model was also based on the optimal balance of complexity and explanatory power by using Akaike’s Information Criterion (AIC) (Akaike, 1987).

## Results

### Twin pair correlations

For the SPHERE psychological distress and somatic distress domain scores at ages 12–15 years, as well as psychological distress at ages 16–19 years, the DZ twin pair correlations were greater than 1/2 of their MZ twin pair counterparts (Supplementary Table S1). This is consistent with familial being aggregation attributable to a combination of additive genetic and shared environmental influences. In contrast, the DZ twin pair correlations at all of the remaining age intervals were 1/2 or less their MZ twin pair counterpart, which is consistent with familial aggregation being attributable to additive genetic and non-additive genetic influences, as described by Falconer (Falconer & Mackay, 1996). In the Classical Twin Design of twins reared together, the effects of non-additivity or dominance (D) and common environmental (C) influences are negatively confounded, and therefore, cannot be modeled simultaneously (Martin et al., 1978a). While distinguishing between D and C effects requires very large samples while also depending on effect sizes and alpha levels, our univariate analyses showed that AE models provided the best fit to the data beyond age 19. Consequently, this empirical evidence, rather than power considerations alone, supported our decision to focus on C rather than D in the univariate and multivariate analyses below.

### Univariate analyses

Detailed univariate model fitting comparisons are shown in Supplementary Table S2. Standardized estimates of the additive genetic (A), shared (C) & unshared (E) environmental influences for each best fitting univariate model are shown in Table 1 (see Supplementary Table S2 for 95% confidence intervals). For somatic and psychological distress at ages 12 to 15 and 16 to 19 years, familial aggregation was explained by a combination of additive genetic and shared environmental influences. At all subsequent ages, familial aggregation was entirely explained by additive genetic influences. From ages 20 to 35 years, the average univariate heritability for psychological and somatic distress ranged from 56% to 68%, respectively.

**Table 1.** Monozygotic (MZ) and dizygotic (DZ) twin pair correlations, standardized additive genetic (A), shared or common environment (C), and non-shared environmental (E) components of variance based on each best fitting univariate model, and longitudinal phenotypic correlations

	Age interval	MZ	DZ	A	C	E	Phenotypic longitudinal correlations					
Somatic distress	12–15 years	0.41	0.27	0.32	0.42	0.26	1					
	16–19 years	0.39	0.16	0.42	0.36	0.22	0.35	1				
	20–23 years	0.32	0.13	0.70	–	0.30	0.15	0.34	1			
	24–27 years	0.32	0.08	0.68	–	0.32	0.21	0.31	0.42	1		
	28–31 years	0.42	0.08	0.70	–	0.30	0.10	0.24	0.36	0.41	1	
	32–35 years	0.37	–0.01	0.65	–	0.35	NA	0.21	0.28	0.51	0.38	1
Psychological distress (anxiety & depression)	12–15 years	0.40	0.27	0.33	0.25	0.41	1					
	16–19 years	0.27	0.15	0.38	0.19	0.43	0.26	1				
	20–23 years	0.32	0.15	0.61	–	0.39	0.22	0.30	1			
	24–27 years	0.30	0.17	0.56	–	0.44	0.17	0.38	0.41	1		
	28–31 years	0.29	0.10	0.53	–	0.47	0.10	0.27	0.36	0.48	1	
	32–35 years	0.34	0.08	0.52	–	0.48	NA	0.33	0.34	0.45	0.46	1

*Note*: All parameter estimates based on full information maximum likelihood, for 95% confidence intervals see Supplementary Table S1, NA = Correlation not estimated since no subjects provided data from both the first (12–15 years) and the last (32–35 years) assessments.

### Longitudinal correlations

Prior to multivariate model fitting we estimated longitudinal phenotypic correlations within each domain. As shown in Table 1, correlations were small to moderately high, ranging from 0.10 to 0.51 (see Supplementary Table S1 for 95% confidence intervals). For somatic and psychological distress, the correlations approached zero as the time between age intervals increased. This simplex structure is consistent with autoregression.

### Multivariate analyses

We then compared the fit of the competing longitudinal models to the saturated correlated factors model (see Table 2). See Supplement for detailed model fitting methods.

**Table 2.** Multivariate model fitting comparisons between the reference correlated factors (null hypothesis) and the competing models. Best fitting models bolded

	Model	ep	−2LL	df	Δ−2LL	Δdf	p	AIC
Somatic distress	Correlated factors	67	18366.74	7223				18500.74
	Dual Change Score	47	18386.74	7243	20.00	20	0.4581	18480.74
	Latent Growth	43	18392.61	7247	25.87	24	0.3595	18478.61
	**Autoregression**	**29**	**18409.83**	**7261**	**43.09**	**38**	**0.2627**	**18467.83**
	1 Common Pathway	34	18452.59	7257	85.85	34	0.0000	18520.59
	2 Common Pathways	46	18389.89	7246	23.15	23	0.4521	18481.89
	3 Common Pathways	61	18375.00	7232	8.26	9	0.5079	18497.00
	Independent Pathways	43	18405.59	7247	38.85	24	0.0283	18491.59
Psychological distress (anxiety & depression)	Correlated factors	67	18782.50	7223				18916.50
	Dual Change Score	47	18800.92	7243	18.42	20	0.5599	18894.92
	Latent Growth	43	18801.19	7247	18.69	24	0.7684	18887.19
	**Autoregression**	**29**	**18812.44**	**7261**	**29.94**	**38**	**0.8215**	**18870.44**
	1 Common Pathway	34	18827.57	7257	45.07	34	0.0971	18895.57
	2 Common Pathways	46	18801.06	7246	18.57	23	0.7262	18893.06
	3 Common Pathways	61	18793.02	7232	10.52	9	0.3099	18915.02
	Independent Pathways	43	18803.53	7247	21.03	24	0.6367	18889.53

*Note*: ep = number of estimated parameters, −2LL = −2 × log-likelihood, Δ−2LL = change in −2 × log-likelihood, Δdf = change in degrees of freedom, AIC = Akaike Information Criteria.

#### Somatic distress

For somatic distress, the dual change, autoregression, and the 2- and 3-factor common pathway models did not deteriorate significantly when compared to the correlated factors. However, the autoregression was chosen as the best fitting based on the lowest AIC values. We next determined if the ‘A’ and ‘C’ autoregression processes could be removed. Neither the shared environmental nor additive genetic influences could be removed from the somatic distress autoregression model (see Supplementary Table S3).

**Figure 2.**
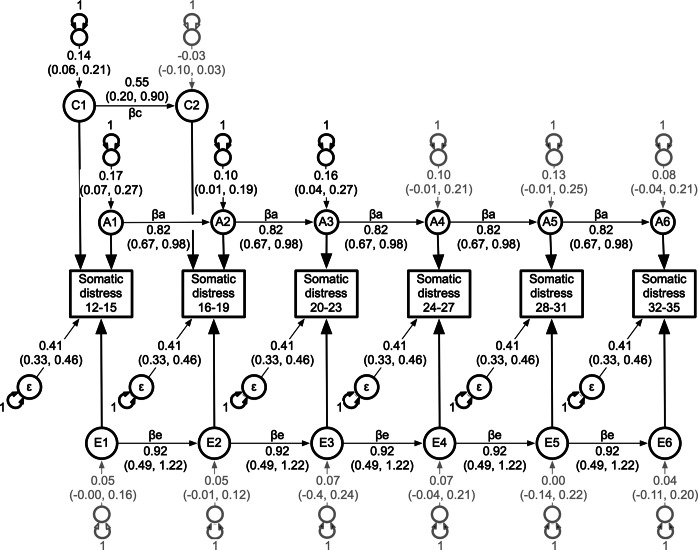
Best fitting multivariate autoregression ACE model for the SPHERE somatic distress scale from ages 12 to 35. *Note:* Illustrated are the latent genetic (A1-A6), shared environment (C1-C2), and non-shared environmental (E1-E6) components and their age-specific genetic, shared environmental, and non-shared environmental innovations, along with transient non-shared environmental influences including measurement error (ε). The genetic, shared, and non-shared environmental autoregression causal coefficients (βa, βc & βe) are each constrained equal across time. 95% confidence intervals are estimated for all free parameters. Age-specific innovation variances are constrained to one, as are factor loadings from each latent ‘A’, ‘C’ and ‘E’ component to their corresponding observed phenotypes. Transient, non-shared environmental influences (ε) are constrained equal across all age intervals for model identification and parsimony.

When based on the lowest AIC value, the multivariate ‘ACE’ autoregression model would normally be selected as the best fit (Table 3). However, as illustrated in Supplementary Figure S3, apart from the first age interval at 12–15 years, the 95% confidence intervals spanning all of the remaining shared environmental innovations (ages 16–35 years) spanned zero. This is consistent with the univariate results, which revealed no significant ‘C’ influences beyond age 19. In post hoc analyses, we, therefore, we dropped from the ‘ACE’ autoregression model the ‘C’ innovations at the last four time intervals (C3–C6), including the last four causal coefficients (βc) starting at age 16 years (see Figure 2). This model provided the best fit to these data (see Table 3).

**Table 3.** Multivariate model fitting comparisons between the ACE autoregression, competing AE, CE and E sub-models, and post hoc analyses for somatic and psychological distress

	Model	ep	-2xLL	df	Δ-2LL	Δdf	p	AIC
Somatic distress	ACE	29	18409.83	7261				18467.83
	AE	22	18424.69	7268	14.86	7	0.0378	18468.69
	CE	22	18457.31	7268	47.48	7	0.0000	18501.31
	E	15	18682.29	7275	272.46	14	0.0000	18712.29
	ACE post-hoc	25	18414.18	7265	4.35	4	0.3603	18464.18
Psychological distress (anxiety & depression)	ACE	29	18812.44	7261				18870.44
	AE	22	18825.27	7268	12.83	7	0.0764	18869.27
	CE	22	18840.41	7268	27.97	7	0.0002	18884.41
	E	15	19038.28	7275	225.84	14	0.0000	19068.28
	ACE post-hoc	25	18818.83	7265	6.39	4	0.1720	18868.83

*Note:* A = additive genetic, C = common or shared environment, E = non-shared environment, ep = number of estimated parameters, -2LL = -2 x log-likelihood, Δ-2LL = change in -2 x log-likelihood, Δdf = change in degrees of freedom, AIC = Akaike Information Criteria. Post-hoc = shared environmental autoregression processes at first two age intervals only (see Figures 2-3).

As illustrated in Figure 2, there were significant genetic innovations at the first three age intervals (A1–A3). All remaining genetic innovations (A3–A6) were non-significant. Despite the significant non-shared environmental autoregression causal coefficients (*β*_e_), all non-shared environmental innovations (E1–E6) were non-significant. In contrast, transient influences, including measurement not captured by this model, were significant at each age interval.

Supplementary Table S3 shows the standardized variance components attributable to additive genetic, shared and non-shared environmental influences, and the additive genetic and non-shared environmental longitudinal correlations based on this best fitting multivariate model. Heritability was moderate and ranged from 22% to 36%, and at ages 12–15 years, there were significant shared environmental correlations that explained 18% of the total variance in somatic distress.

#### Psychological distress

**Figure 3.**
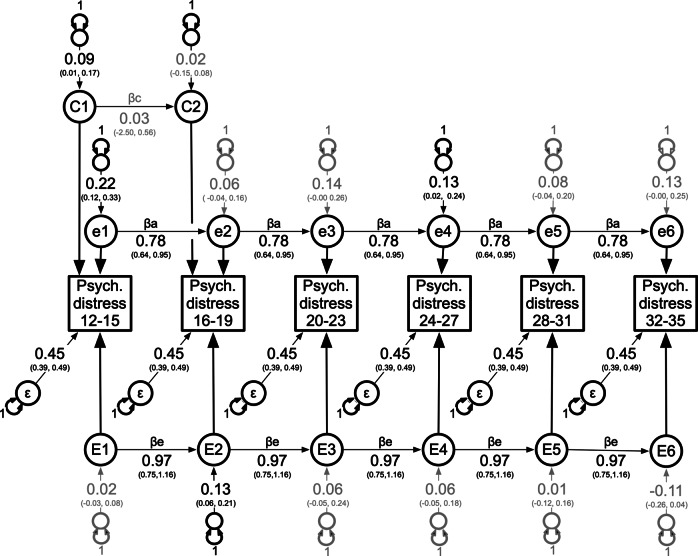
Best fitting multivariate autoregression ACE model for the SPHERE psychological (anxiety and depression) distress scale from ages 12 to 35. *Note:* Illustrated are the latent genetic (A1-A6), shared environment (C1-C2), and non-shared environmental (E1-E6) components and their age-specific genetic, shared environmental, and non-shared environmental innovations, along with transient non-shared environmental influences including measurement error (ε). The genetic, shared, and non-shared environmental autoregression causal coefficients (βa, βc & βe) are each constrained equal across time. 95% confidence intervals are estimated for all free parameters. Age-specific innovation variances are constrained to one, as are factor loadings from each latent ‘A’, ‘C’ and ‘E’ component to their corresponding observed phenotypes. Transient, non-shared environmental influences (ε) are constrained equal across all age intervals for model identification and parsimony.

For psychological distress, all seven competing multivariate models provided a good fit, as judged by the non-significant changes in chi-square. Again, the autoregression was chosen as the best fitting based on the lowest AIC values (see Table 2), but because the 95% confidence intervals spanning the shared ‘C’ environmental innovations from age 16 onwards included zero (see Supplementary Figure S4), we modeled in post hoc analyses, a ‘C’ autoregression process at the first two age intervals only (see Figure 3). This model provided a better fit to these data (see Supplementary Table S3).

As illustrated in Figure 3, there were significant genetic innovations at ages 12–15 and 24–27. The second shared environmental innovation (C2) and causal regression coefficient (*β*_c_) from C1 to C2 were non-significant. With the exception of psychological distress measured at ages 16–19, the non-shared environmental innovations (E1, E3–E6) were non-significant. All remaining variances were explained by transient influences (including measurement error) not captured by the autoregression processes.

Supplementary Table S3 shows the standardized variance components attributable to additive genetic and non-shared environmental influences, as well as the longitudinal additive genetic and non-shared environmental correlations based on this best fitting multivariate model. Heritability was moderate and ranged from 24% to 34%, and at ages 12 to 15 years there were shared environmental influences that explained 11% of the total variance in psychological distress.

## Discussion

Individual differences in self-report, non-clinical measures of psychological and somatic distress from ages 15 to 35 years were best captured by autoregression processes comprising mostly stable genetic influences. For somatic distress, the genetic influences observed at ages 12–15 years were stable and enduring. In addition, time-specific genetic influences emerged at 16–19 and 24–27 years. Significant shared environmental influences were also observed at ages 12–15 years. For psychological distress, the significant genetic influences observed at ages 12–15 years were also stable and enduring. There were also dynamic, age-specific genetic influences observed at ages 24–27 years. Finally, the impact of non-shared environmental influences was largely transient for both anxiety and depression and somatic distress.

Our findings are broadly consistent with previous applications of genetically informative autoregression models to longitudinal measures of anxiety and depression symptoms. Additive genetics account for stability whereas environmental influences are largely short-term and transient (Gillespie et al., 2015; Gillespie, Kirk, et al., 2004; Nivard et al., 2015). Contrary to our results, analyses by Nivard et al. (Nivard et al., 2015) (based on a much larger sample [*N* = 49,524 twins]) found no significant genetic innovations or age-specific genetic influences beyond age 18 years. We found small, but significant genetic innovations at ages 24–27 years. Nivard et al.’s average heritability from ages 12 to 25 was also higher (43.7% versus 31.2%). Such differences between the Australian and Dutch samples may reflect cohort or regional differences or, more likely, differences in the anxiety and depression scales employed. To our knowledge, there are no reports of genetically informative autoregression models to longitudinal measures of somatic distress, which is a common but often ignored dimension of affective disorders (Hansell et al., 2012).

Our results have implications for molecular efforts aimed at identifying alleles associated with symptoms of anxiety, depression and somatic distress. Broadly, the results of autoregression modeling here and elsewhere (Nivard et al., 2015) suggest that estimating polygenic risk scores in young adult or adolescent samples when based on adult meta-analytic genome-wide association scan (GWAS) discovery summary statistics may not be optimal. Wherever biometrical genetic innovations are observed, age-stratified GWAS should be explored and may reveal developmentally specific allelic variants associated with anxiety and depression and somatic distress.

### Limitations

Our results should be interpreted in the context of at least four potential limitations. First, to maximize power we jointly analyzed data from men and women. It is important to recognize that sex can not only affect differences in means, but differences in the causes of residual variance, the latter being our primary interest. For example, in our previous analysis of self-report anxiety and depression symptoms from ages 20 to 70 years we observed different patterns in men and women (Gillespie, Kirk, et al., 2004). For men, genetic variation across all age intervals could be explained by genetic innovations occurring at age 20, indicating longitudinal stability. In contrast, women showed a more complex pattern with age-specific genetic effects emerging at different life stages: at age 30 for anxiety, and at ages 40 and 70 for depression. This suggests that while genetic influences on anxiety and depression are largely stable among men, women experience additional genetic effects that come into play at specific points across the lifespan, which, we speculate, may be attributable to (but not limited to) sex differences in (i) hormonal variation at specific life stages, (ii) gene-environment interactions, (iii) maturation and brain development, and (iv) cumulative stress and social role transitions. In the current analyses, we included sex as a fixed covariate in all our analyses. While this corrects for mean differences, the estimated variance components are pooled across sex, thus obscuring any differences in genetic architectures between men and women. In the best fitting autoregression models, we found that the effect of sex on mean levels of somatic distress was not significant (*β*_sex_ = −0.02, 95% CI = −0.07, 0.03), whereas for psychological distress (anxiety and depression), men reported significantly lower scores (*β*_sex_ = −0.14, 95% CI = −0.08, −0.09). Nivard et al. (2015) also found sex differences in their longitudinal analysis of combined, non-clinical anxiety and depression items. In contrast, Kendler et al.’s (2008) analysis of parental and self-reports on a combined measure of anxiety and depression in men and women aged 8–20 years found no qualitative or quantitative sex differences in genetic influences. Future studies with larger sample sizes could explore sex-specific patterns in these age-dependent genetic innovations. Such studies could test hypotheses related to hormonal changes, gene-environment interactions, and psychosocial factors across different life stages.

Second, our data were limited to ages 12–35 years inclusive. The reports by Nivard et al. (Nivard et al., 2015) and Kendler et al. (Kendler et al., 2008), which included twins as young as 3 and 8 years respectively, found that childhood anxiety and depression were genetically very dynamic. Note that Nivard et al. (2015) applied an autoregression model to longitudinal measures of combined, non-clinical anxiety, and depression items. Data came from maternal reports for ages 3–12 and self-reports for ages 12–18. Similarly, Kendler et al. (2008) collected longitudinal data on combined anxiety and depression from parental and self-reports at ages 8–20, to which they applied a rater bias model to each wave before fitting a Cholesky Decomposition. Genetically informative twin analyses by Silberg et al. (Silberg et al., 2001) have suggested that symptoms of depression measured before and after age 14 years may be etiologically distinct syndromes.

Third, although comprehensive, our modeling was not exhaustive. Kendler et al. found that DSM-III-R major depression was not etiologically homogeneous, but instead was comprised of partially distinct syndromes with different clinical, genetic and longitudinal profiles (Kendler et al., 1996). It was beyond the scope of the current report to test for heterogeneous classes of anxiety and depression or somatic distress.

Finally, our analyses relied on self-report data and may not generalize to clinical diagnoses. For example, we have shown that genetic risks in major depressive disorder (MDD) are not entirely explained by the common factor underpinning the general liability to self-report depression symptoms, Neuroticism and MDD (Kendler et al., 2019). In future analyses, we plan to fit longitudinal models to psychiatric symptoms of MDD.

## Conclusions

We explored the genetic and environmental influences on longitudinal measures of self-report anxiety and depression and somatic distress. We evaluated the fit of the autoregressive, latent growth, dual change, common and independent pathway models to the longitudinal data, with the aim of identifying the model that best describes the observed longitudinal covariance and most accurately accounts for variation and covariation in genetic and environmental influences in anxiety and depression and somatic distress from ages 15 to 35 years. Anxiety and depression including somatic distress were best explained by autoregression modeling implying that an initial quantum of genetic predisposition is carried forward through teenage and early adult development with reasonable fidelity, augmented by small, age-specific genetic innovations. Familial aggregation was entirely explained by additive genetic influences. These influences were largely stable from ages 12 to 35, but also dynamic with evidence of small but significant age-dependent genetic influences. In contrast, environmental influences were largely transient and age-specific. The longitudinal trajectory of anxiety and depression including somatic distress from ages 12 to 35 was best captured by autoregression modeling, which reveals a combination of largely stable as well as smaller dynamic genetic influences.

## Supporting information

Gillespie et al. supplementary material 1 Gillespie et al. supplementary material

Gillespie et al. supplementary material 2 Gillespie et al. supplementary material

## Data Availability

Data are available through requests to N.G. Martin.

## Supplementary material

The supplementary material for this article can be found at http://doi.org/10.1017/S0033291724003222.
