# Supplementary material for: The impact of genes and environment assessed longitudinally on psychological and somatic distress in twins from ages 15 to 35 years
Source: Psychol Med. 2025 Feb 6;55:e17. doi: 10.1017/S0033291724003222 (PMC11968121; doi:10.1017/S0033291724003222)
Supplement: Gillespie et al. supplementary material 1 — Gillespie et al. supplementary material [file S0033291724003222sup001.docx]

**Supplementary information**

**S1. 12-item short version of the Somatic and Psychological HEalth REport (SPHERE).**

When responding, subjects were asked, “*Over the past few weeks have you been troubled by...*?”. Responses were recorded on 3-point ordinal scale: 0 “sometimes/never”; 1 “often”; and 2 “most of the time” before summing.

Psychological distress items:

Feeling nervous or tense

Feeling unhappy and depressed

Feeling constantly under strain

Everything getting on top of you

Losing confidence

Being unable to overcome difficulties

Somatic distress items:

Muscle pain after activity

Needing to sleep longer

Prolonged tiredness after activity

Poor sleep

Poor concentration

Tired muscles after activity

## S2. Multivariate analyses

No subjects provided data at the first (12 to 15 years) and last (32 to 35 years) assessments. We, therefore, constrained the additive genetic (A), shared (C), and non-shared (E) environmental covariances between these age intervals to zero in the correlated factors model.

In order to enable model identification, the 2- and 3-factor common pathway models were fitted using the mxConstraint option to constrain the total variance of each common pathway to one. Therefore, in the 2- and 3-factor common pathway models, there are enough data to calculate specific values for the parameters in each model. Arguably, this may not resolve rotational indeterminacy. One can rotate these factors in different ways, resulting in solutions that are mathematically equivalent. These rotated solutions will explain the same total amount of variance, but they will distribute the variance differently among the factors. Importantly, rotating the factors will not change how well the model fits the data overall.

For the autoregression (AR) model to be identified, either the factor loadings from the latent components to the observed variables must be set to unity and the variance of the innovations estimated, or the variances of the innovation terms need to be standardized to one and the factor loadings estimated. In the present study, we used the first option.

Finally, the variance of the last measurement error parameter in the AR model must be constrained to be equal with at least one of the preceding error terms for the model to be identified. This is because error variance on these occasions would otherwise be indistinguishable from innovation variance. The error parameters will also include variance attributable to short-term non-shared environmental effects. These effects are likely to be different across the lifespan. However, rather than constrain the last two error parameters, our approach was to constrain them to be equal across all age intervals.

A final point concerning the AR model is the distinction between innovations of latent genetic and environmental components and the measurement errors pertaining to observed variables (εi). Although an innovation on a latent component at time i is not caused by the preceding component at time i-1, it nevertheless influences all subsequent time points. This is in contrast to measurement errors, which are terms that do not influence observed variables at subsequent time points. Autoregression designs, therefore, permit the discrimination of transient factors affecting measurement at one time point from factors that are continuously present or exert a long-term influence throughout the time series (Boomsma *et al.*, 1989; Neale & Cardon, 1992).

**Supplementary Figure S1.** Histogram of square root transformed somatic distress (Somdis) symptom counts.

**Supplementary Figure S2.** Histogram of square root transformed psychological distress symptom counts.

**Supplementary Figure S3**


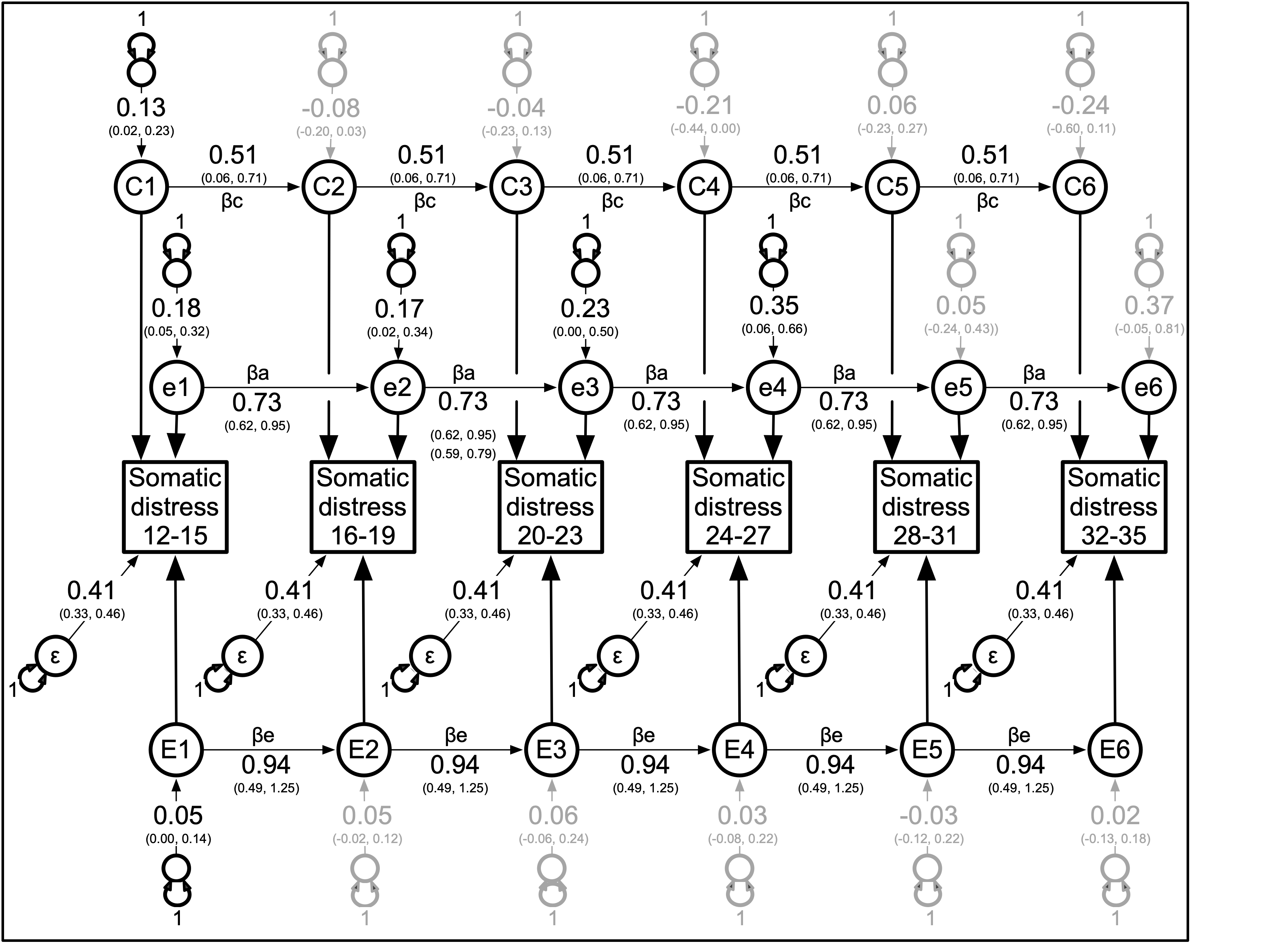


**Note:** Illustrated are the latent genetic (A1-A6), shared environment (C1-C6), and non-shared environmental (E1-E6) components and their age-specific genetic, shared environmental, and non-shared environmental innovations, along with transient non-shared environmental influences including measurement error (ε). The genetic, shared, and non-shared environmental autoregression causal coefficients (βa, βc & βe) are each constrained equal across time. Ninety-five % confidence intervals are estimated for all free parameters. Age-specific innovation variances are constrained to one, as are factor loadings from each latent 'A', 'C' and 'E' component to their corresponding observed phenotypes. Transient, non-shared environmental influences (ε) are constrained equal across all age intervals for model identification and parsimony.

**Supplementary Figure S4**


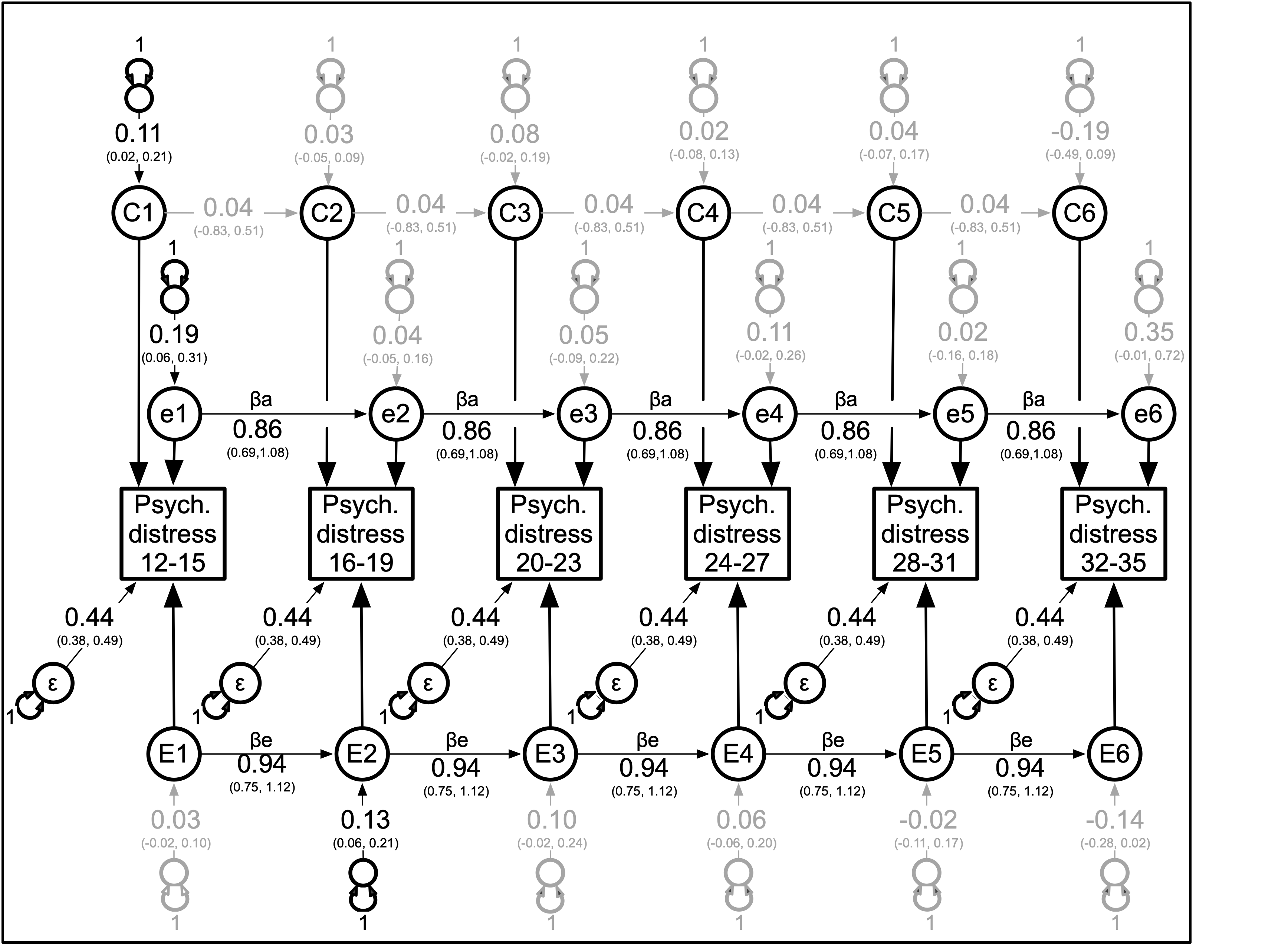


**Note:** Illustrated are the latent genetic (A1-A6), shared environment (C1-C6), and non-shared environmental (E1-E6) components and their age-specific genetic, shared environmental, and non-shared environmental innovations, along with transient non-shared environmental influences including measurement error (ε). The genetic, shared, and non-shared environmental autoregression causal coefficients (βa, βc & βe) are each constrained equal across time. Ninety-five % confidence intervals are estimated for all free parameters. Age-specific innovation variances are constrained to one, as are factor loadings from each latent 'A', 'C' and 'E' component to their corresponding observed phenotypes. Transient, non-shared environmental influences (ε) are constrained equal across all age intervals for model identification and parsimony.
